# Interspecific mating bias may drive *Aedes albopictus* displacement of *Aedes aegypti* during its range expansion

**DOI:** 10.1093/pnasnexus/pgac041

**Published:** 2022-04-14

**Authors:** Jiayong Zhou, Shuang Liu, Hongkai Liu, Zhensheng Xie, Liping Liu, Lifeng Lin, Jinyong Jiang, Mingdong Yang, Guofa Zhou, Jinbao Gu, Xiaohong Zhou, Guiyun Yan, Anthony A James, Xiao-Guang Chen

**Affiliations:** Department of Pathogen Biology, Institute of Tropical Medicine, School of Public Health, Southern Medical University, Guangzhou 510515, China; Department of Pathogen Biology, Institute of Tropical Medicine, School of Public Health, Southern Medical University, Guangzhou 510515, China; Department of Pathogen Biology, Institute of Tropical Medicine, School of Public Health, Southern Medical University, Guangzhou 510515, China; Department of Pathogen Biology, Institute of Tropical Medicine, School of Public Health, Southern Medical University, Guangzhou 510515, China; Guangdong Provincial Center for Disease Control and Prevention, Guangzhou 511430, China; Guangdong Provincial Center for Disease Control and Prevention, Guangzhou 511430, China; Yunnan Provincial Institute of Parasitic Disease Control, Simao 665099, China; Yunnan Provincial Institute of Parasitic Disease Control, Simao 665099, China; Program in Public Health, University of California, Irvine, CA 92697, USA; Department of Pathogen Biology, Institute of Tropical Medicine, School of Public Health, Southern Medical University, Guangzhou 510515, China; Department of Pathogen Biology, Institute of Tropical Medicine, School of Public Health, Southern Medical University, Guangzhou 510515, China; Program in Public Health, University of California, Irvine, CA 92697, USA; Department of Microbiology and Molecular Genetics, University of California, Irvine, CA 92697, USA; Department of Molecular Biology and Biochemistry, University of California, Irvine, CA 92697, USA; Department of Pathogen Biology, Institute of Tropical Medicine, School of Public Health, Southern Medical University, Guangzhou 510515, China

**Keywords:** *Aedes aegypti*, *Aedes albopictus*, interspecific mating, reproductive interference

## Abstract

*Aedes albopictus* is the most invasive mosquito in the world and often displaces *Ae. aegypti* in regions where their populations overlap. Interspecific mating has been proposed as a possible cause for this displacement, but whether this applies across the range of their sympatry remains unclear. *Aedes albopictus* and *Ae. aegypti* collected from allopatric and sympatric areas in China were allowed to interact in cage experiments with different crosses and sex-choices. The results confirm that asymmetric interspecific mating occurs in these populations with matings between allopatric *Ae. albopictus* males and *Ae. aegypti* females being significantly higher (55.2%) than those between *Ae. aegypti* males and *Ae. albopictus* females (27.0%), and sympatric mosquitoes showed a similar but lower frequency bias, 25.7% versus 6.2%, respectively. The cross-mated females can mate second time (remate) with the respective conspecific males and the 66.7% remating success of female *Ae. albopictus* was significantly higher than the 9.3% of *Ae. aegypti* females. Furthermore, 17.8% of the matings of *Ae. albopictus* males exposed to mixed pools of *Ae. albopictus and Ae. aegypti* females and 9.3% of the matings of *Ae. aegypti* males with mixed *Ae. aegypti* and *Ae. albopictus* females were interspecific. The difference in the length of clasper between male *Ae. albopictus* (0.524 mm) and *Ae. aegypti* (0.409 mm) may be correlated with corresponding mates. We conclude that stronger *Ae. albopictus* male interspecific mating and more avid female intraspecific remating result in a satyr effect and contribute to competitive displacement of *Ae. aegypti* as allopatric *Ae. albopictus* invade during range expansion.

Significance Statement *Aedes albopictus* and *Ae. aegypti* are highly invasive mosquitoes and important vectors of arboviruses that cause disease in humans, which make them major public health threats. Both species have sympatric and allopatric populations and appear to be in competition where they overlap with *Ae. albopictus* often displacing *Ae. aegypti*. We show that there is sex-based, asymmetric interspecific mating between the two species in China that is characterized by stronger interspecific mating of male *Ae. albopictus* and higher intraspecific remating by female *Ae. albopictus* that may drive *Ae. albopictus* displacement of *Ae. aegypti* during its range expansion. These findings confirm and extend previous studies on interspecific competition between the two species and could benefit novel mosquito control methods and prevent mosquito-borne diseases.

## Introduction

*Aedes aegypti* is the primary vector of dengue, Zika, and yellow fever viruses and is distributed mainly in the global tropical regions (1–3). *Aedes albopictus* is the most important vector of chikungunya virus and also can transmit dengue viruses. Furthermore, it is the most invasive mosquito species worldwide and is distributed widely in tropical, subtropical, and temperate regions. It is now found in all continents except Antarctica (4–6). *Aedes* mosquitoes and their transmitted viral pathogens represent major threats to public health (7).

*Aedes aegypti* and *Ae. albopictus* share similar life cycles and ecological niches and often are found distributed sympatrically (8, 9). *Aedes aegypti* was found in the state of Florida in the United States, since 1880 (10). When *Ae. albopictus* invaded the state in the 1980s (11, 12), it gradually replaced *Ae. aegypti* as the local dominant species (13, 14), even causing total displacement in some regions (15–17). Similarly, *Ae. aegypti* used to be the predominant species during the 1980s in Hainan, China (18) and caused several dengue fever pandemics on the island and in the neighboring Leizhou peninsula, a joint area among Guangdong, Hainan, and Guangxi Provinces (19). At the time, *Ae. albopictus* was found rarely on the island. Several recent studies report that *Ae. aegypti* is now rare in the region and that *Ae. albopictus* is widespread (20). Both *Ae. aegypti* and *Ae. albopictus* have been on the Leizhou peninsula since the 1980s (21). Since then, the population and density of *Ae. aegypti* has been decreasing in this area and now only small numbers are found in the limited regions of Wushi town and Qishui town of Zhanjiang city, and there is a trend of *Ae. albopictus* completely replacing *Ae. aegypti* in the near future (22). Similar trends of species replacement also have been reported in the southeastern United States (23, 24) and Bermuda (25), where *Ae. albopictus* displaced *Ae. aegypti* with comparable rapidity. It is unclear how *Ae. albopictus* replaces *Ae. aegypti* although competition for breeding sites, and asymmetries in interspecific mating and the effects of male accessory gland proteins have been proposed (24, 26–28).

Satyrization is a form of mating interference in which males of one species mate with females of another species, produce no hybrid progeny, and significantly decrease the reproductive fitness of the species from which the females originate (27, 29, 30). Satyrization has been proposed as the probable cause of competitive displacements of resident mosquitoes by invasive species, especially of *Ae. aegypti* by *Ae. albopictus* (24). Cage experiments and field observations indicated that *Ae. albopictus* males are capable of satyrizing females of other species of the Stegomyia subgenus, potentially leading to competitive displacements, and possible extinctions, especially of endemic species on islands (27). However, the general dynamics of interspecific matings between *Ae. albopictus* and *Ae. aegypti* are not known.

Collections of *Aedes albopictus* and *Ae. aegypti* from allopatric and sympatric regions in China were used to investigate the dynamics of satyrization (Figure S1 and Table S1, Supplementary Material). Interspecific and intraspecific matings and rematings between *Ae. albopictus* and *Ae. aegypti* show a strong bias for favoring *Ae. albopictus* in regions where they might overlap with *Ae. aegypti*. These data confirm and extend what was seen with populations in Florida, USA (24, 31).

## Results

### Asymmetric interspecific matings occur at different frequencies in laboratory- and field-derived *Ae. albopictus* and *Ae. aegypti* in China

**Fig. 1.**
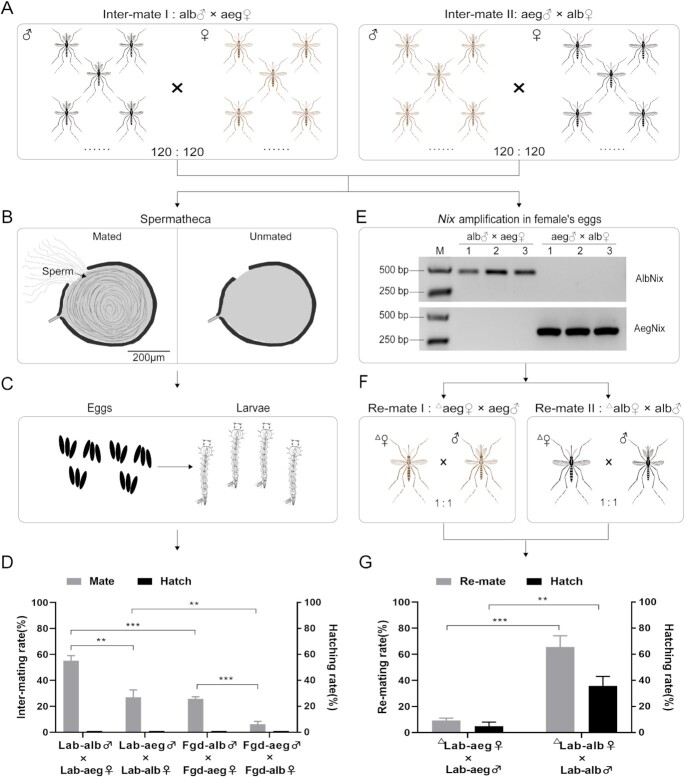
Interspecific matings and outcomes between *Ae. albopictus* and *Ae. aegypti*. (A) Intermate I: male *Ae. albopictus* × female *Ae. aegypti*, Intermate II: male *Ae. aegypti* × female *Ae. albopictus*. A total of 120 males and 120 females were exposed in a cage for 7 days. (B) Female spermathecae were dissected to determine their mating status. (C) Eggs and hatched larvae per female mosquito were recorded after interspecific exposure. (D) Interspecific mating and hatching rates between *Ae. albopictus* and *Ae. aegypti*. Lab: laboratory strain; Fgd: field Guangdong strain. (E) Eggs per female mosquito were detected for male specific *Nix* gene by PCR to confirm whether the mosquito had been interspecific mated or not. (F) Remate I: intermated female *Ae. aegypti* × male *Ae. aegypti*, Remate II: intermated female *Ae. albopictus* × male *Ae. albopictus*. “△” represents females that had previously mated interspecifically. In total, one interspecific mated female was exposed to one conspecific male in a cup for 5 days, then laid eggs and hatching situation from every female mosquito were recorded. (G) Remating and hatching rate between intermated females and conspecific males. The black mosquito icons represent *Ae. albopictus* and brown mosquito icon represents *Ae. aegypti*. Gray columns represent intermating or remating rate; black columns represent hatching rate. Bars represent the standard error of mean. Statistics were performed using Student t test. ***P < 0.001 and **P < 0.01. These assays were repeated three times.

A total of two interspecific mating groups for both laboratory- and field-collected mosquitoes were examined. Intermating I consists of male *Ae. albopictus* crossed with female *Ae. aegypti* and Intermating II has male *Ae. aegypti* crossed with female *Ae. albopictus*. In total, two control mating groups also were done: Control I had male *Ae. albopictus* crossed with female *Ae. albopictus*, and Control II had male *Ae. aegypti* crossed with female *Ae. aegypti*. Interspecific mating was observed in both groups (Fig. 1; Table S2, Supplementary Material). The interspecific mating rate for the laboratory mosquitoes was significantly higher in group I (55.2 ± 2.2%) compared to group II (27.0 ± 3.3%; *t* = 7.15, d.f. = 4, and *P* = 0.0020). This asymmetry also was observed and significant in the sympatric field-collected mosquitoes, 25.7 ± 1.0% and 6.2 ± 1.3% for Intermatings I and II, respectively, (*t* = 12.07, d.f. = 4, and *P* = 0.0003) but was less frequent. These data support the conclusion that interspecific matings between *Ae. albopictus* and *Ae. aegypti* are asymmetric, with *Ae. albopictus* males having a much higher rate of successful intermating than *Ae. aegypti* males. Furthermore, the higher interspecific mating rates between allopatric laboratory populations and sympatric Guangdong field-derived populations (Student t tests, all *P* < 0.01) support the hypotheses that prolonged sympatry may lead to selection for more robust premating barriers between the two species, especially for *Ae. aegypti* (32).

### Interspecific-mated female mosquitoes do not produce viable offspring

The overall oviposition rates were high for females in all mating groups (χ^2^ = 6.51, d.f. = 5, and *P* = 0.0852), regardless of being unmated or mated interspecifically or intraspecifically (Table S3, Supplementary Material). However, the number of eggs per female varied among mating groups, and the egg-hatching rates among all cross-species-mated were zero, and consequently, no progeny were produced. The control intraspecific egg-hatching rates were 74.1% to 79.4% and these combined data support and confirm previous reports that interspecies matings in either direction do not result in viable progeny (Fig. 1) (24).

### Remating between interspecific mated females and conspecific males

Remating experiments between previously interspecific mated females and conspecific males were carried out only for the laboratory-reared mosquito colonies. As expected, remating rates were low, 9.3 ± 1.0%, for *Ae. aegypti* for which it is known that the first mating inhibits subsequent mating (33, 34) (Fig. 1; Table S4, Supplementary Material). *Aedes albopictus* rematings were significantly higher,66.7 ± 4.2%, (*t* = 11.24, d.f. = 4, and *P* = 0.0004) and the odds ratio of remating was 25.6 (95% CI: 7.3 to 90.1) for *Ae. albopictus* over *Ae. aegypti*. Similarly, the hatching rate of eggs produced by those previously cross-species mated females was significantly highly for *Ae. albopictus* (35.8 ± 4.2%) compared to *Ae. aegypti* (4.9 ± 1.8%; *t* = 6.78, d.f. = 4, and *P* = 0.0025). These data support the conclusion that cross-mated female *Ae. albopictus* and *Ae. aegypti* can remate with their conspecific males with the former being more likely to contribute to the subsequent generation. However, a basal difference in remating rates between the species could explain these observed differences (35).

### Male *Ae. albopictus* and *Ae. aegypti* mate differentially with conspecific and interspecific females in a competition assay

**Fig. 2.**
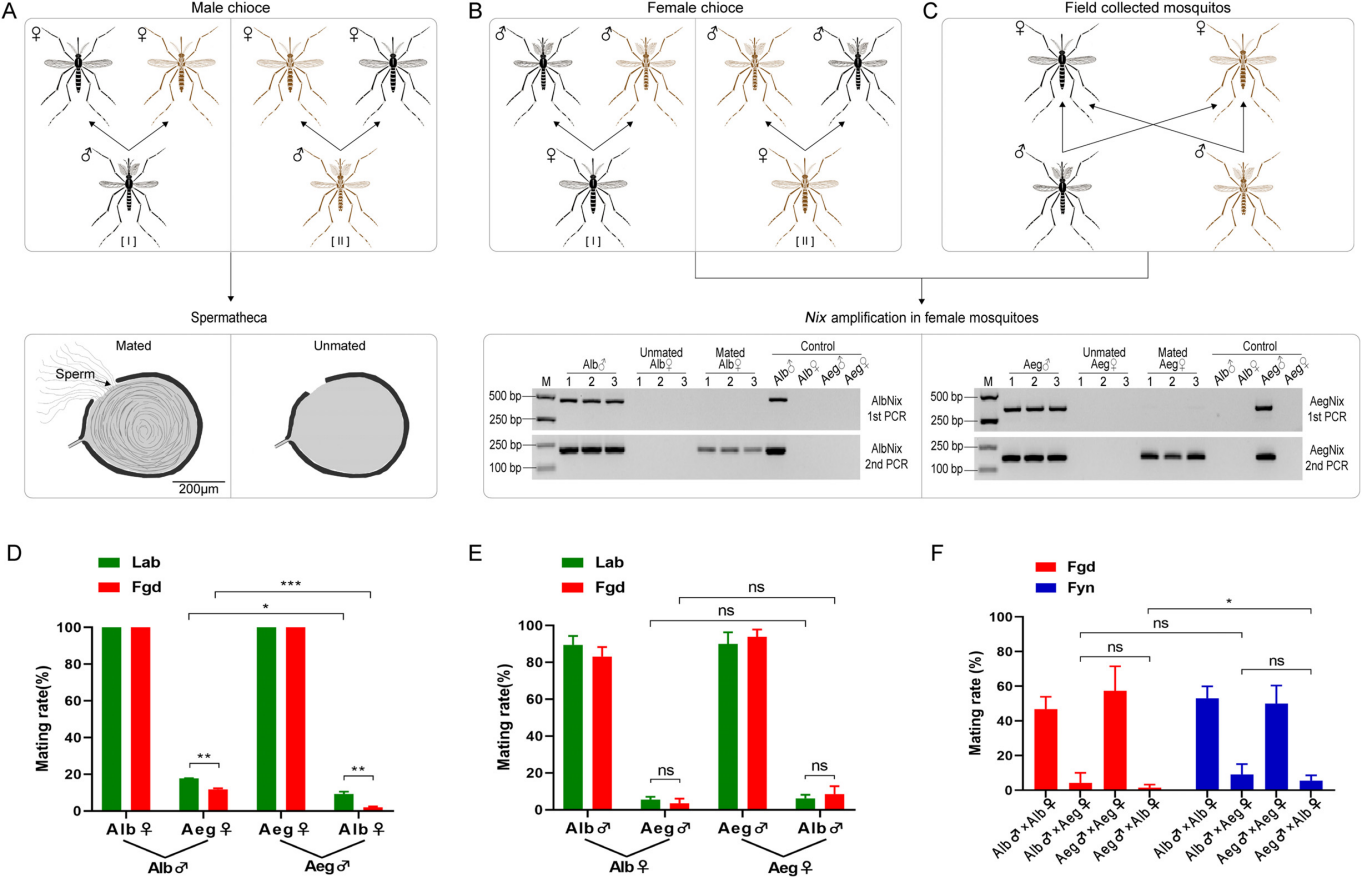
Choice-mating among *Ae. albopictus* and *Ae. aegypti*. (A) Male-choice I: 100 male *Ae. albopictus* × (100 female *Ae. albopictus* + 100 female *Ae. aegypti*), Male-choice II: 100 male *Ae. aegypti* × (100 female *Ae. aegypti* + 100 female *Ae. albopictus*). Those males and females were exposed in a cage for 7 days, then female spermathecae were dissected to determine their mating status. (B) Female-choice I: 100 female *Ae. albopictus* × (100 male *Ae. albopictus* + 100 male *Ae. aegypti*), Female-choice II: 100 female *Ae. aegypti* × (100 male *Ae. aegypti* + 100 male *Ae. albopictus*). Those females and males were exposed in a cage for 7 days, then female mosquitoes were used to detect male specific *Nix* gene by Nest PCR to identify whether the female had been intraspecific or interspecific mated. (C) Choice mating in field mosquitoes. Both *Ae. albopictus* and *Ae. aegypti* mosquitoes collected in the field were used to detect male specific *Nix* gene by Nest PCR to identify whether the female had been intraspecific or interspecific mated. (D) Male-choice mating rates between *Ae. albopictus* and *Ae. aegypti*. (E) Female-choice mating rates between *Ae. albopictus* and *Ae. aegypti*. (F) Intraspecific and interspecific mating rates of *Ae. albopictus* and *Ae. aegypti* collected from the fields. The black mosquito icons represent *Ae. albopictus* and brown mosquito icon represents *Ae. aegypti*. Lab: laboratory stain (green columns). Fgd: field Guangdong strain (Zhanjiang; red columns). Fyn: field Yunnan strain (Jinghong; blue columns). (D)–(E) Bars represent standard error of mean, and statistics were performed using Student t test. (F) Bars represent 95% CI, and statistics were performed using χ^2^-test or Fisher exact test. *P < 0.05, **P < 0.01, ***P < 0.001, and ns = not significant. Male- and female-choice mating assays were repeated three times. The field mosquitoes were collected five times in Zhanjiang, Guangdong and Jinghong, Yunnan, respectively.

The results of a series of two mating experiments (“male-choice”), *Male-alb*, comprising *Ae. albopictus* males crossed with mixed *Ae. albopictus* and *Ae. aegypti* females, and *Male-aeg, Ae. aegypti* males crossed with *Ae. aegypti* and *Ae. albopictus* females, revealed interspecific mating rates of 17.8 ± 0.1% and 9.3 ± 1.3% (*t* = 5.25, d.f. = 3, and *P* = 0.0135), respectively, for laboratory populations and 11.8 ± 0.6% and 2.0 ± 0.4% (*t* = 13.77, d.f. = 4, and *P* = 0.0002), respectively, for Guangdong field-derived populations (Fig. 2; Table S5, Supplementary Material). Control intraspecific mating rates were 100%. Male *Ae. albopictus* had 2- to 5-fold higher proportion of interspecific matings compared to male *Ae. aegypti*, and the allopatric populations (laboratory colonies) were higher than the sympatric populations (Guangdong field-derived). These findings support the conclusion that male mosquito choice is a significant factor in interspecific matings and is consistent with prolonged sympatry selecting for more robust premating barriers (31, 32).

### Female-choice does not play a major role in interspecific matings between *Ae. albopictus* and *Ae. aegypti*

Similar experiments with females (“female choice”) *Female-alb*, in which *Ae. albopictus* females were crossed with *Ae. albopictus* and *Ae. aegypti* males, and *Female-aeg*, crosses of *Ae. aegypti* females with *Ae. aegypti* and *Ae. albopictus* males, showed similar intraspecific mating rates, ranging from 83.2 ± 5.2% to 93.9 ± 3.9% (Fig. 2; Table S6, Supplementary Material). Furthermore, the interspecific mating rates also were similar, ranging from 3.5 ± 2.5% to 8.5 ± 4.3% (Student t tests, all *P* > 0.05). These results support the conclusion that female choice of either *Ae. albopictus* or *Ae. aegypti* does not play a major role as a premating barrier for subsequent interspecific mating.

### Evidence of interspecific mating between *Ae. albopictus* and *Ae. aegypti* in field-derived mosquitoes

Examination of field-collected adult females showed intraspecific mating rates ranging from 46.9 ± 3.6% to 57.5 ± 7.2% for *Ae. albopictus* and *Ae. aegypti*, respectively (χ^2^ tests, all *P* > 0.05; Fig. 2; Table S7, Supplementary Material). In contrast, the interspecific mating rates between male *Ae. albopictus* and female *Ae. aegypti* or male *Ae. aegypti* and female *Ae. albopictus* were 4.3 ± 2.9% and 1.6 ± 0.9%, respectively, in Zhanjiang samples, and 9.1 ± 3.1% and 5.5 ± 1.6%, respectively, in Jinghong samples. Only the matings biases of *Ae. aegypti* males with *Ae. albopictus* females between Zhanjiang and Jinghong were significant (*P* < 0.05). Interestingly, *Ae. albopictus* and *Ae. aegypti* have been sympatric in Zhanjiang for more than 40 years (21) but less than 10 years in Jinghong (36), and these data are consistent with the hypothesis that prolonged sympatry selects for premating barriers between these two species (32, 37).

### *Aedes albopictus* males have longer claspers than *Ae. aegypti* males

**Fig. 3.**
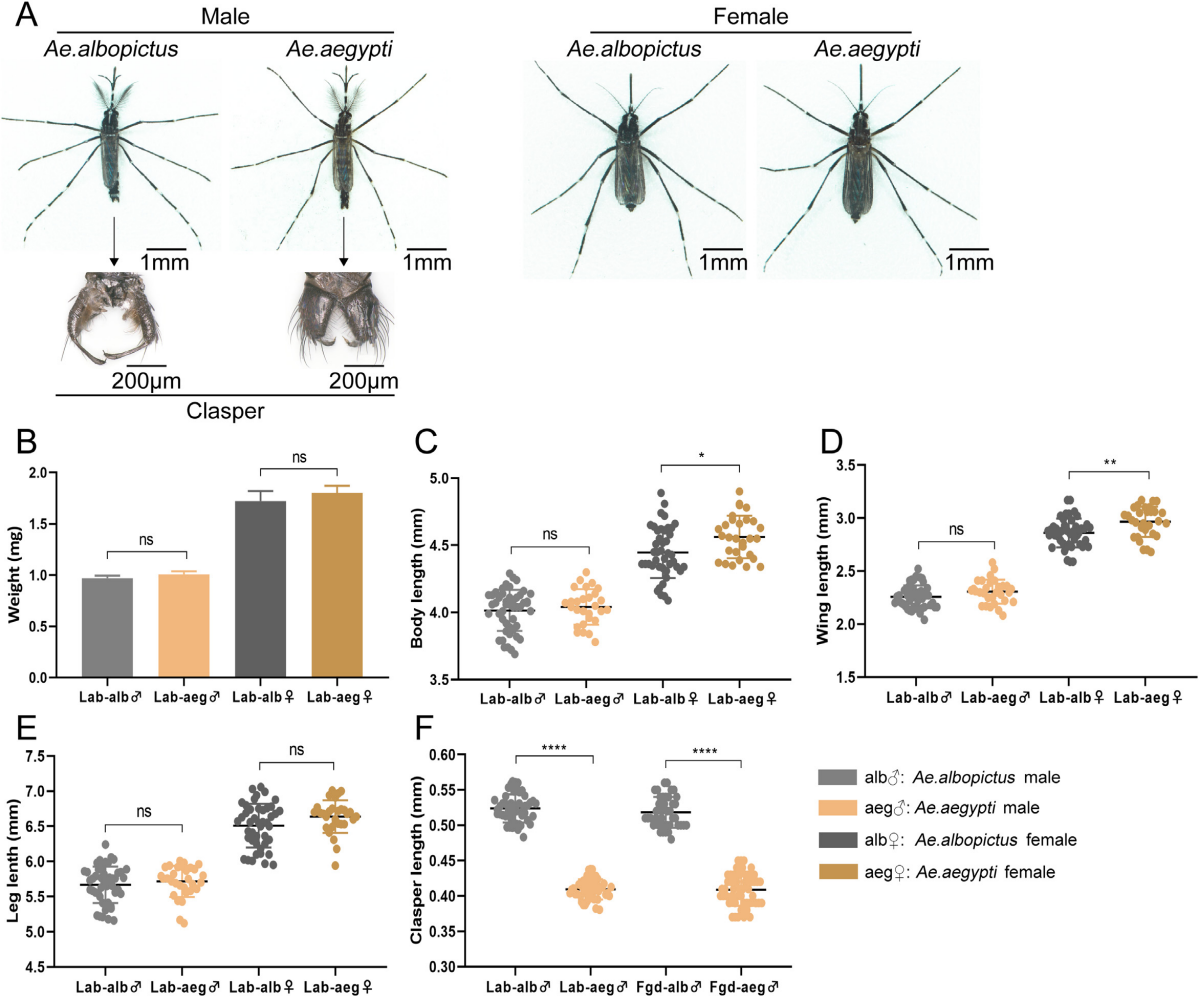
Morphological comparison and physical measurements of *Ae. albopictus* and *Ae. aegypti*. (A) Morphology and male clasper of *Ae. albopictus* and *Ae. aegypti*. (B) Weight of adult mosquitoes (mg). (C) Body length of adult mosquitoes (mm). (D) Wing length of adult mosquitoes (mm). (E) Leg length of adult mosquitoes (mm). (F) Clasper length of male mosquitoes (mm). Lab: laboratory strain. Fgd: field Guangdong strain. alb♂:*Ae. albopictus* male (light gray); aeg♂: *Ae. aegypti* male (light brown); alb♀:*Ae. albopictus* female (dark gray); aeg♀: *Ae. aegypti* female (dark brown). Bars represent standard error of mean. Statistics were performed using Student t test. *P < 0.05, **P < 0.01, ****P < 0.0001, and ns = not significant. A total of 10 mosquitoes were assigned to each group. The measurements were repeated five times.

Morphological characteristics may be among the factors contributing to the observed differentials in interspecific matings between *Ae. albopictus* and *Ae. aegypti*. While female mosquitoes are generally larger than their conspecific males, the sizes and coloration of both male and female *Ae. albopictus* and *Ae. aegypti* are similar (Fig. 3; Table S8, Supplementary Material). The average weight, body length, wing length, and leg length in samples of 50 each male and female mosquitoes also are similar. In contrast, the average length of the clasper in male *Ae. albopictus* is 0.524 ± 0.003 mm and 0.518 ± 0.003 mm in laboratory and Zhanjiang field-derived mosquitoes, respectively, and these are significantly longer than those of male *Ae. aegypti* (laboratory strain 0.409 ± 0.002 mm;  field mosquitoes 0.409 ± 0.003 mm; Student t tests, all *P* < 0.0001).

### *Aedes albopictus* copulation bouts are of longer duration than those of *Ae. aegypti*

**Fig. 4.**
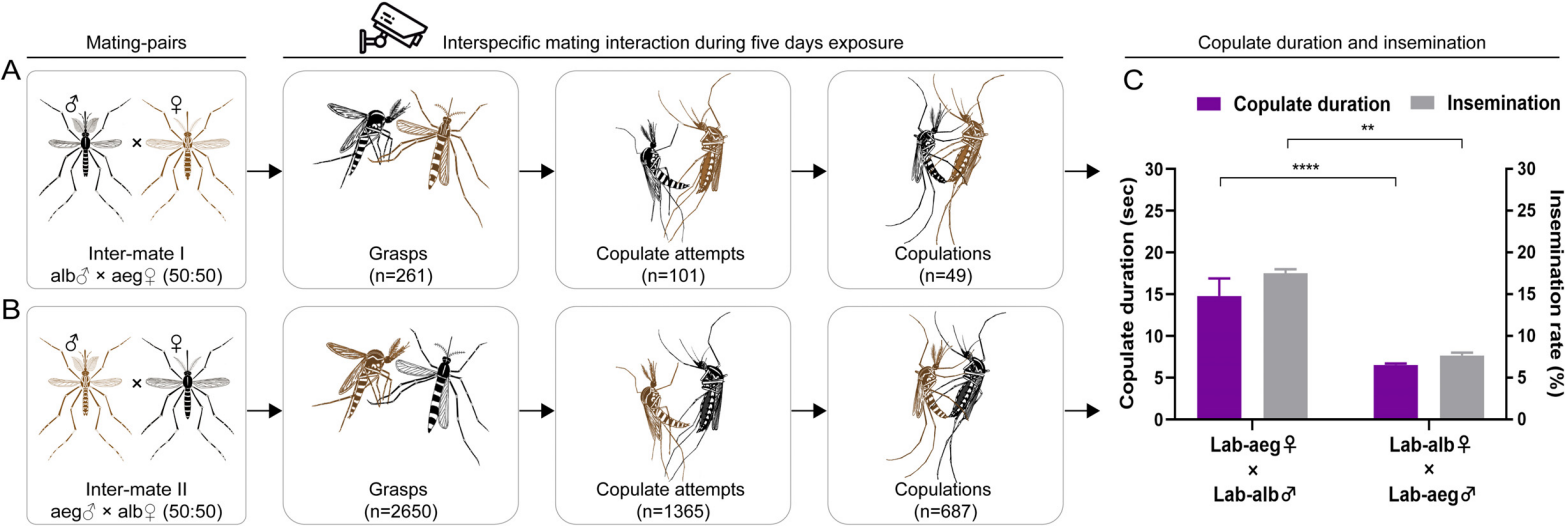
Video observations of interspecific mating interactions between *Ae. albopictus* and *Ae. aegypti*. (A) Interspecific mating interaction of Intermate I (alb♂ × aeg♀); (B) Interspecific mating interaction of Intermate II (aeg♂ × alb♀). A total of 50 males and 50 heterospecific females were transferred into a custom video cage. The videos were recorded at intervals from ZT0-3 (3 h after light on) and ZT11-14(3 h before light off) for five consecutive days. Grasp: male grasps female; Copulate attempt: male rolls its abdomen to try copulate; Copulation: male copulates with female successfully; (C) Copulate duration and insemination rate of intermate I and intermate II. Copulate duration: the average time from copulation to separation (s); Insemination rate = (number of females with sperm/number of dissected females) × 100%. The black mosquito icons represent *Ae. albopictus* and brown mosquito icon represents *Ae. aegypti*. Purple columns represent copulate duration; Gray columns represent insemination rate. Bars represent standard error of mean. Statistics were performed using Student t test. **P < 0.01 and ****P < 0.0001. The video observations were repeated twice.

Video imaging of interspecific mating bouts of Intermate I: *Ae. albopictus* males with *Ae. aegypti* females and Intermate II: *Ae. aegypti* males with *Ae. albopictus* female revealed that the grasping episodes (*n* = 261; males attempting to engage females) of *Ae. albopictus* males with *Ae. aegypti* females were significantly lower than *Ae. aegypti* males against *Ae. albopictus* females (*n* = 2650), indicating that *Ae. aegypti* males were more active in pursuit of a heterospecific females than male *Ae. albopictus* (Fig. 4; Table S9, Supplementary Material). The copulating attempts, successful copula, and copulate-fail rates were 101, 49, and 51.49%, respectively, in Intermate I, and were 1,365, 687, and 49.67%, respectively, in Intermate II. These data support the conclusion that the acceptances of both female mosquito species to the interspecific males are similar and the females do not play the dominant role in mate choice. Interestingly, the copulating duration and insemination rate in Intermate I was 14.78 ± 2.12 s and 17.54 ± 0.47%, which are significantly longer and higher than 6.54 ± 0.19 s and 7.66 ± 0.34% in Intermate II (Student t tests, all *P* < 0.01). The copulating duration in control I (*Ae. albopictus* males with *Ae. albopictus* females) was 20.60 ± 0.95 s,  which also significant longer than 7.55 ± 0.32 s (Student t tests, *P* < 0.0001) in control II (*Ae. aegypti* males with *Ae. aegypti* females; Figure S3 and Table S9, Supplementary Material). These results are consistent with interspecific mating rates and coincident with male clasper lengths between *Ae. albopictus* and *Ae. aegypti*.

## Discussion

The results of these experiments demonstrate that asymmetric interspecific matings can occur between *Ae. albopictus* and *Ae. aegypti* mosquito samples collected in China, and these findings are consistent with studies in southeastern United States (24, 38, 39) and Bermuda (25). The interspecific matings between *Ae. albopictus* males and *Ae. aegypti* females were significantly more frequent than the reciprocal matings of *Ae. aegypti* males with *Ae. albopictus* females. Interspecific matings also were more frequent in allopatric strains than in sympatric strains. Furthermore, females that had mated to interspecific males produced no progeny. This combination of results provide a basis for the observed gradual displacement of *Ae. aegypti* by *Ae. albopictus* in Zhanjiang. From these results, we conclude that *Ae. albopictus* suppresses the reproduction of *Ae. aegypti* by interspecific mating and this is an example of satyrization in species competition. These observations extend the conclusions of the previous findings to include large-scale arenas of invasion and displacement in China.

The insemination rate is typically used to assess male performance in mosquitoes (40–42), but the role of female choice and the degree to which it influences mating outcomes is not known (43, 44). The interspecific mating frequencies in male choice groups observed in this study were significantly different and *Ae. albopictus* males mated more frequently with *Ae. aegypti* females, even in the presence of conspecific females, than *Ae. aegypti* males did with *Ae. albopictus* females under similar choice conditions. In contrast, no significant differences were observed in the female choice experiments. These findings support the conclusion that interspecific matings between *Ae. albopictus* and *Ae. aegypti* is dominated by male behavior, and male *Ae. albopictus* engage in interspecific mating more frequently than male *Ae. aegypti*. While the evidence for female choice in mating bias is not evident here, it may arise as a premating barrier as the species experience longer periods of sympatry (32, 37). This asymmetric mating coupled with the infertility of interspecific-mated females would have a significant impact on species displacement.

Male mating behavior and morphology may provide a partial explanation for the asymmetrical interspecific mating bias. One correlated factor is that the average length of the clasper in *Ae. albopictus* males is significantly longer than that of *Ae. aegypti* males. The claspers are used to hold the female tightly during copulation and prevent easy disengagement during sperm transfer, and therefore, result in a more successful mating. Longer copulation times and increased insemination rates of *Ae. albopictus* male compared with *Ae. aegypti* were observed and detected in video experiments. We do not propose that these morphological differences alone account for the male-driven asymmetry in mating success, but they may have an additive effect in combination with other factors including sex pheromones (45) and wing-beat frequency (46, 47). Further studies are needed to clarify these and other potential mechanism for the asymmetric interspecific matings.

Female mosquitoes usually mate only once in their reproductive lifetime (48–50) because the sperm from the first mating can be stored in spermatheca and used throughout subsequent gonotropic cycles (51). In addition, substances secreted by the accessory glands and passed to the female along with the sperm alter female mating behavior to prevent remating (33, 52). However, we showed here that *Ae. albopictus* or *Ae. aegypti* females previously experiencing an interspecific mating could remate with a conspecific male and produce viable offspring. It is note-worthy that the remating and hatching rates of *Ae. albopictus* females were significantly higher than those of female *Ae. aegypti*. This is likely due to the differential effectiveness of male accessory gland protein suppression of remating in the interspecific crosses (24). The accessory protein HP-1 from the semen of *Ae. albopictus* could impose enforced monogamous paternity on *Ae. aegypti* females and inhibit secondary matings, but in contrast, secretions of *Ae. aegypti* males did not have the effect on female *Ae. albopictus* (53). Other factors correlated with the female activity in remating possibly exist in the semen of the male (33, 54) and need to be clarified with more study.

*Aedes albopictus* and *Ae. aegypti* share similar life cycle characteristics and ecological habits (8, 55, 56), as well as the similar size, body weight, and wing length. As the result, both mosquitoes also may have similar swarm behavior, wing beat frequencies, and other premating factors that make possible occasional interspecific mating. Because the reproductive cost of interspecific mating is high, no viable progeny, resistance to interspecific mating would be expected to be selected against during divergence of the ancestral mosquitoes that give raise to these two species (32, 37). Consistent with this expectation, interspecific matings among field-derived Zhanjiang samples where *Ae. albopictus* and *Ae. aegypti* have been sympatric for at least 40 years were significantly lower than those samples where both mosquitoes have only been sympatric for less than 10 years. Similar observations have been reported for these two species in sympatric locales in Florida, USA (32, 37). It is possible that more prolonged close contact of both mosquitoes leads to stronger premating barriers, so that the errant interspecific matings could be avoided and decreased. This observed asymmetry also may account for the circumstances where *Ae. albopictus* as a species invading regions where *Ae. aegypti* is already extant leads to the suppression and elimination of the latter.

## Conclusions

We can conclude from these studies that the asymmetric interspecific matings between *Ae. albopictus* and *Ae. aegypti* characterized by *Ae. albopictus* males exhibiting more frequent interspecific mating than *Ae. aegypti* males, and female *Ae. albopictus* having a greater frequency of conspecific remating is a general phenomenon in areas where these mosquitoes are sympatric. This results in a species competition known as satyrization in which *Ae. albopictus* can displace *Ae. aegypti*. This asymmetry is correlated with the length of clasper and female monogamy. These findings highlight some of the potential factors and mechanism of interspecific mating and species competition between *Ae. albopictus* and *Ae. aegypti*, two important vector and invasive species. Furthermore, the observed biases may complement and enhance the efficacy of sterile insect technologies (SIT) for impacting pathogen transmission dynamics in regions where the two species are sympatric and able to transmit the same pathogens.

## Materials and Methods

### Mosquito strains and rearing

Laboratory *Ae. albopictus* and *Ae. aegypti* strains have been colonized in our laboratory for many years. Field-derived colonies of Zhanjiang *Ae. albopictus* and *Ae. aegypti* were collected from artificial containers (discarded tires and buckets) in several places in Wushi Town, Zhanjiang City, Guangdong Province in 2019 to 2021, and these colonies used in the mating experiments were second to fourth generation (F_2_ to F_4_). All mosquitoes were reared at 27 ± 1°C,  70 ± 10% humidity and under 14 h light/10 h dark cycles. To obtain experimental mosquitoes, the larvae (200 larvae/l water) were reared in stainless steel trays containing dechlorinated water and were provided daily with yeast and turtle food. Pupae of both species were collected individually and secured in 2 ml Eppendorf tubes with 1 ml water. When adults emerged, the species and sex of adults were determined by examination of the scutum and antennae, respectively. Adults of each sex and species were placed separately in paper bowls (9.5 × 6.7 × 6.2 cm^3^) with a mesh cover and offered a 10% sucrose solution on a cotton wick.

### Noncompetitive (no-choice) interspecific mating experiments

Experiments were conducted in microcosms (20 × 20 × 30 cm^3^) using virgin males and females of each species, originating from the laboratory colonies and the field-derived colonies of Guangdong Province. All mosquitoes were between 2- and 3-d-old when used for mating experiments. A total of 120 males of one species was crossed with 120 unmated females of the other species in each enclosure (microcosm) using laboratory (lab) or field-derived mosquitoes. Mosquitoes were left to cohabit for 7 days. Conspecific microcosms containing males and females of either *Ae. albopictus* and *Ae. aegypti* were set up as normal mating controls. In addition, unmated females of each species were held alone in microcosms as egg-laying controls. All females were anesthetized with CO_2_ and spermathecae dissected to determine insemination status. The presence of sperm in spermathecae was recorded as an insemination event (Figure S2, Supplementary Material). Triplicate replicates were carried out for every cross combination.

### Gene amplification (PCR) to detect the male*-*specific *gene, Nix*, in mated females

*Nix* is a male-specific gene present in both *Ae. albopictus* (57) and *Ae. aegypti* (58) and can be used to detect mated females. If female mosquitoes mated, the sperm of male mosquitoes would be transferred to the spermathecae of female mosquitoes. PCR assays were used to detect *Nix* in eggs to determine whether females had mated. Moreover, we established a sensitive and specific nested PCR assay to detect *Nix* in female mosquitoes to identify whether the female had been intraspecifically or interspecifically mated. The sequences of primers and program used in the study are given in Table S10 (Supplementary Material). *AlbNix* and *AegNix* were used to detect *Nix* in eggs as well as the first fragment in female mosquitoes, and Nest-*AlbNix* and Nest-*AegNix* were used to amplify the second fragment which is inside of the first fragment. For the first run, female genomic DNA was used for template. For the second run, the first run of PCR product was diluted 100 times as a template. Positive and negative controls were included in each first run experiment. Amplification products were observed under UV light after electrophoresis in 1% agarose gel containing and confirmed by DNA sequencing.

### Remating experiment

The remating assays recapitulated the noncompetitive (no-choice) experiment described above. Females from the intercross experiments were offered a blood meal after a week of expose to the interspecific males. Restrained mice were put in the cages, in compliance with the recommendations in the Guide for the Care and Use of Laboratory Animals of the NIH. Individual engorged females were transferred to a 250-ml paper cup with filter paper. Females were given 3 days to lay eggs. The pools of eggs were counted and the DNA extracted used as a template for gene amplification to detect the presence of *Nix* gene. Only mosquitoes positive for *Nix*-eggs were used for remating to ensure female had indeed mated with an interspecific male. These females then were transferred to a paper cup and mated for 5 days with conspecific males at a ratio of 1:1. Blood fed again. Every blood-fed female was transferred to a new paper cup with filter paper. Eggs collected from each female were counted and hatched to determine if a female deposited viable or nonviable eggs. Production of viable eggs was used as a proxy for successful intraspecific insemination. In the controls, 15-d-old virgin females were exposed to 3-d-old conspecific males for 5 days and subsequently allowed to blood feed. Each blood-fed female was allowed to oviposit. There were 10 to 20 females per replicate and three repetitions were performed.

### Male-choice and female-choice mating experiment

A total of 100 males were aspirated into microcosms (20 × 20 × 30 cm^3^) containing 100 conspecific and 100 heterospecific females in the male-choice experiment. They were left to cohabit for 7 days. After that, the females were removed and morphologically identified as *Ae. albopictus* and *Ae. aegypti*. The spermatheca of female mosquitoes then were dissected and examined the sperm microscopically. A total of 100 females were aspirated into microcosms containing 100 conspecific and heterospecific males for the female-choice experiments. After 7 days, females were removed. Each female mosquito was transferred into a 1.5-ml Eppendorf tube containing 50 μl of lysis buffer from MiniBEST Universal Genomic DNA Extraction Kit (Takara-Bio, Shiga, Japan). The mosquito was digested by proteinase K and RNase A overnight at 56°C according to the manufacturer's instructions. Following DNA extraction, samples were stored at −20°C until PCR analysis.

### Detection of cross-mating in natural populations

Adult mosquitoes were collected from randomly selected locales in two regions in Southern and Southwestern China, where *Ae. albopictus* and *Ae. aegypti* coexist to determine potential interspecific matings in natural populations. Human landing catches were carried out using power aspirators at two different cities in China. Wushi town (109°86“E, 20°56” N), located in the southwest of Leizhou Peninsula, is one of the main fishing ports in Guangdong Province. The other city, Jinghong (101°31“E,22°36”N), is located in the southern part of Yunnan Province and adjacent to Myanmar. Both cities have larval habitats for *Ae. albopictus* and *Ae. aegypti*. Wild-caught females were identified morphologically as *Ae. albopictus* and *Ae. aegypti* and were stored in ethanol. They were transferred to a plastic plate and washed three times in deionized water to remove ethanol. In order to further determine which male mosquitoes the females mated with, *Nix* (*Ae. albopictus, AlbNix* and *Ae. aegypti*, and*AegNix*) diagnostic fragments were amplified from their genomic DNA using the previously described nested PCR system.

### Measurement of adult mosquito

The weight, body length, wing length, and leg length of adult male and female *Ae. albopictus* and *Ae. aegypti* (laboratory strains), as well as the length of the male mosquito clasper (laboratory and field-derived Guangdong strains) were measured. Mosquitoes were placed in an oven to dry for 1 h, and their weight was measured in groups of 10 mosquitoes (repeated five times). Forceps and dissecting scissors were used to separate the legs, wings, and body of mosquitoes and the male mosquito claspers. These were examined microscopically and images recorded using a computer and camera. Image-pro Plus software was used to measure the length of the organs. Measurements of 50 mosquitoes of each species and sex were taken.

### Video observation of interspecific mating interactions

Newly emerged 2- to 3-d-old mosquitoes of laboratory strains were used for mating behavioral video recording. A total of 50 males and 50 females were transferred into a custom video cage (7 × 11 × 12 cm^3^) and a digital camera (Logitech Capture, 1080P, 60fps) was used to record the mating process between *Ae. albopictus* and *Ae. aegypti*. The videos were recorded at intervals from ZT0-3 (3 h after light on) and ZT11-14 (3 h before light off) for five consecutive days. The number of grasp (male grasps female), copulation attempt (male rolls its abdomen to try copulate), copulation (male copulates with female successfully), copulation fail rate, and mean of copulate duration and insemination rate were observed and counted.

### Statistical analysis

All statistical analyses were performed using SPSS version 20.0 (IBM, Chicago, IL). Mating rates, remating rates, hatching rates, mosquito eggs, body size, and male clasper length were compared using the Student t test (significant level of *α* = 0.05). χ^2^-test or Fisher exact test (if any *n* < 5) (significant level of *α* = 0.05) was used to compare the mating rate in wild-caught female mosquitoes. The odds ratio of remating success (after interspecific mating) was calculated for *Ae. albopictus* against *Ae. aegypti*.

\begin{eqnarray*} {\rm{Odds \, ratio:}} & \ {\rm{Percentage\, deviation\, is\, calculated\, as }}\\ & \frac{{Observed - expercted}}{{expected}} \times 100. \end{eqnarray*}

## Supplementary Material

pgac041_Supplemental_File

## Data Availability

All relevant data are within the manuscript and its Supplementary Information files
