# Supplementary material for: Interspecific mating bias may drive Aedes albopictus displacement of Aedes aegypti during its range expansion
Source: PNAS Nexus. 2022 Apr 14;1(2):pgac041. doi: 10.1093/pnasnexus/pgac041 (PMC9112929; doi:10.1093/pnasnexus/pgac041)
Supplement: pgac041_Supplemental_File [file pgac041_supplemental_file.docx]

**Supplementary Information for**

Interspecific mating bias may drive *Aedes albopictus* displacement of *Aedes aegypti* during its range expansion

Jiayong Zhou^1#^, Shuang Liu^1#^, Hongkai Liu^1^, Zhensheng Xie^1^, Liping Liu^2^, Lifeng Lin^2^, Jinyong Jiang^3^, Mingdong Yang^3^, Guofa Zhou^4^, Jinbao Gu^1^, Xiaohong Zhou^1^, Guiyun Yan^4^, Anthony A. James^5,6^, Xiao-Guang Chen^1^*

* Corresponding author : Xiao-Guang Chen, Department of Pathogen Biology, Institute of Tropical Medicine, School of Public Health, Southern Medical University, Guangzhou, China, 510515

**Email:**  xgchen@smu.edu.cn

^#^ Contributed equally.

**This PDF file includes:**

Figures S1 to S3

Tables S1 to S10


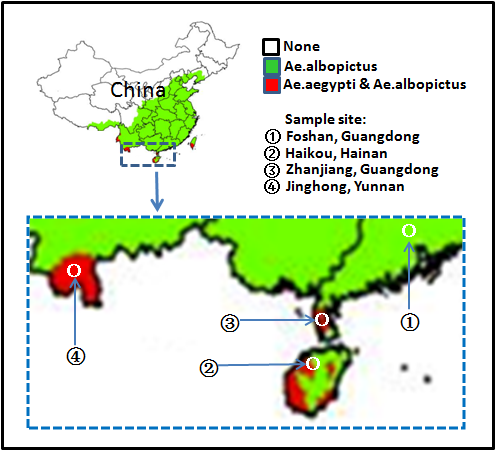


Figure S1.The distribution and sampling sites of *Aedes albopictus* and *Ae. aegypti* in China.


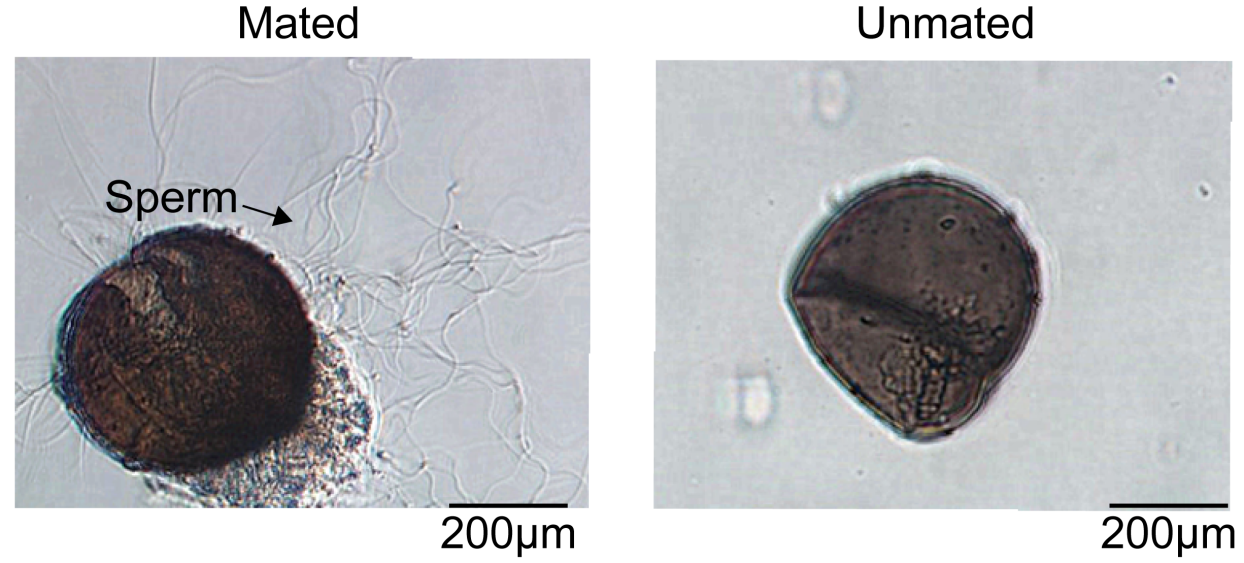


Figure S2. The spermatheca of mated and unmated female mosquito. The arrow points to the sperm.


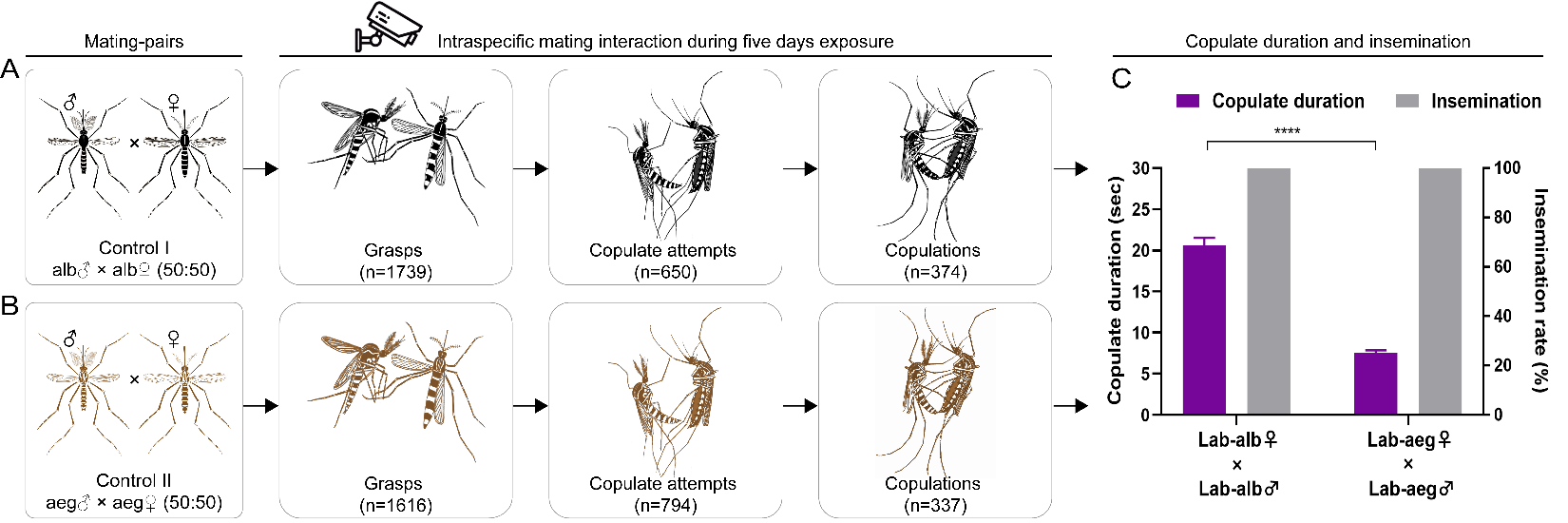


**Figure S3. Video observations of intraspecific mating interactions of control groups.** (A)Intraspecific mating interaction of control I (alb♂×alb♀). (B) Intraspecific mating interaction of control II (aeg♂×aeg♀). 50 males and 50 females were transferred into a custom video cage. The videos were recorded at intervals from ZT0-3 (3 h after light on) and ZT11-14 (3 h before light off) for five consecutive days. (C)Copulate duration and insemination rate of control I and control II. The black mosquito icons represent Ae. albopictus and brown mosquito icon represents Ae. aegypti. Purple columns represent copulate duration; Gray columns represent insemination rate. Bars represent standard error of mean. Statistics were performed using Student t test. *****p* < 0.0001. The video observations were repeated twice.

**Table S1. Experimental strains and annotation of *Aedes albopictus* and *Ae. aegypti.***

| Site | Mosquito | Annotation |
| --- | --- | --- |
| ① | Lab-alb♂  Lab-alb♀ | *Ae. albopictus* was isolated in 1981 in Foshan of Guangdong Province, China, then was kept in insectarium and did not come in contact with *Ae. aegypti.* |
| ② | Lab-aeg♂  Lab-aeg♀ | *Ae. aegypti* was isolated in 2004 in Haikou of Hainan Province, China, then was kept in insectarium and did not come in contact with *Ae. albopictus*. |
| ③ | Fgd-alb♂  Fgd-alb♀ | *Ae. albopictus* was isolated in 2019-2021 in the field of Zhanjiang City of Guangdong Province, China, then was kept in insectarium or extracted DNA for PCR. |
|  | Fgd-aeg♂  Fgd-aeg♀ | *Ae. aegypti* was isolated in 2019-2021 in the field of Zhanjiang City of Guangdong Province, China, then was kept in insectarium or extracted DNA for PCR. |
| ④ | Fyn-alb♂  Fyn-alb♀ | *Ae. albopictus* was isolated in 2019-2021 in the field of Jinghong City of Yunnan Province, China, then was kept in insectarium or extracted DNA for PCR. |
|  | Fyn-aeg♂  Fyn-aeg♀ | *Ae. aegypti* was isolated in 2019-2021 in the field of Jinghong City of Yunnan Province, China, then was kept in insectarium or extracted DNA for PCR. |

Table S2. Non-competitive (no-choice) interspecific matings between *Aedes albopictus* and *Ae. aegypti.*

| Population | Group | Mating-pairs | Dissected females (n) | Mating rate (%,±SEM) |
| --- | --- | --- | --- | --- |
| Laboratory | Ⅰ | alb♂ × aeg♀ | 240 | 55.20±2.21 |
|  |  | alb ♂ × alb♀ | 50 | 100.00 |
|  | Ⅱ | aeg♂ × alb♀ | 302 | 26.97±3.27 |
|  |  | aeg♂ × aeg♀ | 49 | 100.00 |
| Field Guangdong | Ⅰ | alb♂ × aeg♀ | 226 | 25.69±0.98 |
|  |  | alb ♂ × alb♀ | 32 | 100.00 |
|  | Ⅱ | aeg♂ × alb♀ | 251 | 6.18±1.29 |
|  |  | aeg♂ × aeg♀ | 35 | 100.00 |

Note: Shown are the raw data from the mating assays presented in Fig.1. A total of 120 *Ae. albopictus* and 120 *Ae. aegypti* were mated for seven days.Spermathecaes were dissected and examined. The interspecific mating assays were repeated three times. The intraspecific mating assays were repeated twice times.

Table S3. Oviposition status, mean egg per female and egg hatch rate of laboratory strain females.

| Mating group | Females tested (n) | Oviposition rate (%,±SEM) | No. of eggs per female (Mean±SEM) | Hatching  Rate (%,±SEM) |
| --- | --- | --- | --- | --- |
| alb♂ × aeg♀ | 298 | 96.02±1.68 | 91.93±1.52 | 0.00 |
| aeg♂ × alb♀ | 275 | 75.65±2.66 | 57.89±1.31 | 0.00 |
| aeg♀ alone | 147 | 82.29±4.21 | 53.08±2.47 | 0.00 |
| alb ♀ alone | 108 | 84.24±1.22 | 48.57±2.42 | 0.00 |
| aeg ♂×aeg♀ | 161 | 98.74±0.17 | 94.99±2.24 | 79.38±4.33 |
| alb ♂×alb♀ | 162 | 99.39±0.62 | 81.89±1.85 | 74.07±2.99 |

Note: Shown are the raw data from the mating assays presented in Fig.1. A total of 120 *Aedes albopictus* and 120 *Ae. aegypti* were mated for seven days. Oviposition rate, the number of eggs per female and hatching rate were counted. The interspecific mating groups were repeated three times. The no mating groups and intraspecific mating groups were repeated twice times.

**Table S4. Re-mating between interspecific mated females and conspecific males.**

| Group | Mating-pairs | Inter-mated females(n) | Egg-laying females(n) | Fertile females(n) | Re-mating rate (%,±SEM) | No. of total eggs (n) | No. of total larvae (n) | Hatching rate (%,±SEM) |
| --- | --- | --- | --- | --- | --- | --- | --- | --- |
| Ⅰ | ^△^Lab-aeg♀ × Lab-aeg♂ | 42 | 42 | 4 | 9.30±0.99 | 2892 | 155 | 4.91±1.80 |
| Ⅱ | ^△^Lab-alb♀ × Lab-alb♂ | 28 | 28 | 18 | 66.73±4.18 | 1850 | 624 | 35.83±4.19 |

Note: Shown are the raw data from the re-mating assays presented in Fig.1. To confirm whether female mosquitoes were interspecific mating, mosquito eggs were used to detect male specific gene *Nix*. One interspecific mated female was exposed subsequently to one conspecific male. The number of egg-laying females, fertile females, re-mating rate, the number of total eggs and larvae, hatching rate were counted. ‘△’ represents females that had interspecifically mated. The re-mating rate is the number of females with progeny / number of females laying eggs. The assays were repeated three times.

**Table S5. Male-choice interspecific mating between *Aedes albopictus* and *Ae. aegypti.***

| Population | Group | Mating-pairs | Intraspecific mating | |  | Interspecific mating | |
| --- | --- | --- | --- | --- | --- | --- | --- |
|  |  |  | Dissected females (n) | Mating rate（%,±SEM） |  | Dissected females (n) | Mating rate（%,±SEM） |
| Laboratory | Ⅰ | alb♂ × (alb♀ + aeg♀) | 202 | 100.00 |  | 261 | 17.80±0.09 |
|  | Ⅱ | aeg♂ × (aeg♀ + alb♀) | 195 | 100.00 |  | 215 | 9.27±1.26 |
| Field Guangdong | Ⅰ | alb♂ × (alb♀ + aeg♀) | 153 | 100.00 |  | 271 | 11.80±0.59 |
|  | Ⅱ | aeg♂ × (aeg♀ + alb♀) | 215 | 100.00 |  | 206 | 2.03±0.39 |

Note: Shown are the raw data from the male-choice interspecific mating assays presented in Fig.2. Male-choice: 100 *Ae. albopictus* or 100 *Ae. aegypti* males were exposed to 100 conspecific and 100 heterospecific females in the same cage for seven days.Spermathecaes were dissected, and the mating rates were counted. Assays were repeated three times.

**Table S6. Female-choice interspecific mating between *Aedes albopictus* and *Ae. aegypti.***

| Population | Group | Mating-pairs | Detected females(n) | Intraspecific mating only(n) | Interspecific mating only(n) | Mated both(n) | Intraspecific mating (%,±SEM) | Interspecific mating (%,±SEM) |
| --- | --- | --- | --- | --- | --- | --- | --- | --- |
| Laboratory | Ⅰ | alb♀ × (alb♂ + aeg♂) | 180 | 152 | 1 | 9 | 89.44±4.84 | 5.55±1.47 |
|  | Ⅱ | aeg♀ × (aeg♂ + alb♂) | 180 | 152 | 1 | 10 | 90.00±6.31 | 6.11±2.00 |
| Field Guangdong | Ⅰ | alb♀ × (alb♂ + aeg♂) | 155 | 123 | 2 | 4 | 83.17±5.17 | 3.52±2.49 |
|  | Ⅱ | aeg♀ × (aeg♂ + alb♂) | 155 | 134 | 1 | 13 | 93.89±3.89 | 8.45±4.33 |

Note: Shown are the raw data from the female-choice interspecific mating assays presented in Fig.2. Female-choice: 100 *Ae. albopictus* or 100 *Ae. aegypti* females were exposed to 100 conspecific and 100 heterospecific males in the same cage for seven days, then *Ae. albopictus* or *Ae. aegypti* male-specific gene *Nix* was detected in female mosquitoes to confirm whether this female was mating with conspecific or heterospecific males. Assays were repeated three times.

**Table S7. Detected intra- and inter-specific mating between *Aedes albopictus* and *Ae. aegypti* in field mosquitoes.**

| Region | Species | Collected females (n) | Intraspecific mating only(n) | Interspecific mating only(n) | Mated both(n) | Intraspecific mating (%,±SE) | Interspecific mating (%,±SE) |
| --- | --- | --- | --- | --- | --- | --- | --- |
| Zhanjiang, Guangdong | *Ae. albopictus* | 194 | 90 | 2 | 1 | 46.91±3.58 | 1.55±0.89 |
|  | *Ae. aegypti* | 47 | 26 | 1 | 1 | 57.45±7.21 | 4.26±2.94 |
| Jinghong, Yunnan | *Ae. albopictus* | 200 | 98 | 3 | 8 | 53.00±3.53 | 5.50±1.61 |
|  | *Ae. aegypti* | 88 | 42 | 6 | 2 | 50.00±5.33 | 9.09±3.06 |

Note: Shown are the raw data from the interspecific mating between *Aedes albopictus* and *Ae. aegypti* in the field assays presented in Fig. 2 in which *Ae. albopictus* or *Ae. aegypti* females were collected in [sympatric](C:/Users/44558/AppData/Local/youdao/dict/Application/8.9.9.0/resultui/html/index.html#/javascript:;) regions (Zhanjiang, Guangdong and Jinghong, Yunnan). *Ae. albopictus* or *Ae. aegypti* male-specific gene *Nix* was detected in female mosquito to confirm whether this female was mating with conspecific or heterospecific males. The field mosquitoes were collected five times in Zhanjiang, Guangdong and Jinghong, Yunnan respectively.

**Table S8. Measurement of body size and male gonostyli (clasper) of *Aedes albopictus* and *Ae. aegypti.***

| Sample | Species (sex) | Weigh (mg,±SEM） | Body length (mm,±SEM） | Wing length (mm,±SEM） | Leg length (mm,±SEM) | Clasper length (mm,±SEM) |
| --- | --- | --- | --- | --- | --- | --- |
| Laboratory | *Ae. albopictus*♂ | 0.97±0.01 | 4.02±0.02 | 2.26±0.02 | 5.67±0.04 | 0.524±0.003 |
|  | *Ae. aegypti*♂ | 1.01±0.01 | 4.04±0.02 | 2.31±0.02 | 5.72±0.04 | 0.409±0.002 |
|  | *Ae. albopictus*♀ | 1.72±0.04 | 4.45±0.03 | 2.86±0.02 | 6.51±0.05 | —— |
|  | *Ae. aegypti*♀ | 1.80±0.03 | 4.56±0.03 | 2.97±0.03 | 6.64±0.04 | —— |
| Zhanjiang, Guangdong | *Ae. albopictus*♂ | —— | —— | —— | —— | 0.518±0.003 |
|  | *Ae. aegypti*♂ | —— | —— | —— | —— | 0.409±0.003 |

Note: Shown are the raw data from the measurement of body size and male gonostyli (clasper) of *Aedes albopictus* and *Ae. aegypti* presented in  Fig. 3. After standardized feeding, the weight, body length, wing length, leg length and male gonostyli (clasper) of *Ae. albopictus* and *Ae. aegypti* were measured by Image-pro Plus software. 10 mosquitoes were assigned to each group. The measurements were repeated five times.

**Table S9.** **Video observations of interspecific and intraspecific mating interactions between *Aedes albopictus* and *Ae. aegypti* of laboratory strain.**

| Group | Mating pairs | Male grasps female | |  | Male copulates with female | | | | | Insemination rate (%,±SEM) |
| --- | --- | --- | --- | --- | --- | --- | --- | --- | --- | --- |
|  |  | Grasp(n) | Initial grasp time within 3h (hh:mm:ss) |  | Copulate attempt(n) | Copulation(n) | Copulate failure (%) | Initial copulate time within 3h (hh:mm:ss) | Copulate duration (sec,±SEM) |  |
| Inter-mate I | alb♂×aeg♀ | 261 | 0:25:55 |  | 101 | 49 | 51.49 | No copulation | 14.78±2.12 | 17.54±0.47 |
| Inter-mate II | aeg♂×alb♀ | 2650 | 0:01:42 |  | 1365 | 687 | 49.67 | 0:01:53 | 6.54±0.19 | 7.66±0.34 |
| Control I | alb♂×alb♀ | 1739 | 0:00:08 |  | 650 | 374 | 42.46 | 0:00:09 | 20.60±0.95 | 100 |
| Control II | aeg♂×aeg♀ | 1616 | 0:00:08 |  | 794 | 337 | 57.56 | 0:00:12 | 7.55±0.32 | 100 |

Note: Shown are the raw data from the video observations of interspecific mating interactions between *Aedes albopictus* and *Ae. aegypti* presented in  Fig. 4 and Fig.S3. A total of 50 males and 50 females were introduced into a custom video cage. The videos were recorded at intervals from ZT0-3 (3 h after lights-on) and ZT11-14(3 h before lights-off) for five consecutive days. The number of grasp (male grasps female), the number of copulate attempt (male rolls its abdomen to try copulate), the number of copulation(male copulates with female successfully), copulate fail rate, the initial grasp plus copulation time within 3h, the mean of copulate duration and insemination rate were counted. Initial grasp plus copulation time within 3 hours: the time when the first grasp and copulation was observed within 3 hours after mosquitoes were transferred to the video cage on the first day. The video observations were repeated twice times.

**Table S10. Primers and programs for PCR of the male-specific *Nix* gene of *Aedes albopictus* and *Ae. aegypti.***

| Primer name | Sequences （5’-3’） | Annealing temperature and cycles | Usage of primer |
| --- | --- | --- | --- |
| *AlbNix* F | TGCCACACAAACCTTGAAAA | 55 °C, 30 | PCR of *AlbNix* for female’s eggs samples |
| *AlbNix* R | TTCAATCGGTTTTAATAGTTCTGC |  |  |
| Nest-*AlbNix* F | GCTGCAAGGACCATTCTGGA | 56 °C, 25 | Nested PCR of *AlbNix* for female mosquitos |
| Nest-*AlbNix* R | CGTGCAGTTTTGCTTCTGTGA |  |  |
| *AegNix* F | GATTTTTGTTTTTGTCGTGCAA | 55 °C, 30 | PCR of *AegNix* for female’s eggs samples |
| *AegNix* R | AATGCAAGTATCATAGGTCAGCA |  |  |
| Nest-*AegNix* F | GAAATGCTGAGATCAATGCAGAA | 56 °C, 25 | Nested PCR of *AegNix* for female mosquitos |
| Nest-*AegNix* R | AGGTTTAACGTCGCAATGTGG |  |  |
